# CBD promotes antitumor activity by modulating tumor immune microenvironment in HPV associated head and neck squamous cell carcinoma

**DOI:** 10.3389/fimmu.2025.1528520

**Published:** 2025-05-22

**Authors:** Prakriti Sen, Sayed Sadat, Koji Ebisumoto, Riyam Al-Msari, Sayuri Miyauchi, Souvick Roy, Pardis Mohammadzadeh, Kristin Lips, Takuya Nakagawa, Robert Saddawi-Konefka, Andrew B. Sharabi, Joseph A. Califano

**Affiliations:** ^1^ Gleiberman Head and Neck Cancer Center, Moores Cancer Center, University of California San Diego, La Jolla, CA, United States; ^2^ Department of Otolaryngology—Head and Neck Surgery, Moores Cancer Center, University of California San Diego Health, La Jolla, CA, United States; ^3^ Department of Radiation Medicine and Applied Sciences, Moores Cancer Center, University of California, San Diego, La Jolla, CA, United States

**Keywords:** HPV-positive HNSCC, cannabinoid, MAPK, CD4+T cells, CD8+T cells, anti-tumor immune response

## Abstract

**Introduction:**

Marijuana use is associated with HPV-positive head and neck squamous cell carcinoma (HNSCC). However, cannabinoid use continues to increase in the US general population for recreational purposes as well as in cancer patients for palliative care. In this study, we explored the role of cannabidiol (CBD) in promoting anti-tumor activity by modulating immune response in HPV-positive HNSCC by using pre-clinical models.

**Methods:**

The anti-proliferative effect of CBD on HPV-positive HNSCC cells was evaluated through BrdU, apoptosis and migration analyses, followed by western blot analysis to assess its role in activating the MAPK pathway. Next, the anti-tumor immune response of CBD was evaluated in immunocompetent syngeneic mouse as well as in immune-deficient B6.129S7-Rag1^tm1Mom^/J or Rag 1 Knockout mice (Rag1 -/-) and athymic nude mouse. Immune cell infiltration was measured by flow cytometry, IHC and multiplex IHC analysis after subcutaneous injection of mEER cells. Furthermore, the anti-tumor activity of CBD on the tumor microenvironment was evaluated after the depletion of CD4+T cells and CD8+T cells in murine models.

**Results:**

We observed CBD treatment inhibited cell proliferation and migration by promoting apoptosis in HPV-positive HNSCC cells through activation of the MAPK pathway and its associated markers like ERK1/2, JNK/SAPK and MK2. CBD significantly inhibited tumor growth in immunocompetent mice but had no effect in immune-deficient models, indicating an immune-dependent mechanism. CBD enhanced infiltration of CD4+T and CD8+T cells, CD19+B cells, NK cells, and M1-like macrophages into the primary tumors of immunocompetent syngeneic mice models, implicating an enhanced anti-tumor immune response. Interestingly, we observed a significant increase in tumor volume in CD4-depleted mice treated with CBD as compared to CBD-treated wild-type mice suggesting the importance of CD4+T cells in CBD-mediated anti-tumor activity. Finally, multiplex IHC analysis demonstrated co-localization of CD4+T and CD8+ T cells with the activated MAPK marker phospho-p38 in CBD-treated tumors.

**Discussion:**

CBD inhibits tumor cell proliferation in HPV-positive HNSCC by activating the MAPK pathway. It also enhances anti-tumor activity by modulating the tumor immune microenvironment, promoting co-localization of p38 MAPK-activated CD4+ and CD8+ T cells.

## Graphical Abstract

Represents **(A)** HPV positive Head and Neck Squamous Cell Carcinoma mainly arising from the oropharynx region. **(B)** CBD activating the MAPK pathway in HPV positive HNSCC cells followed by decrease in cell proliferation, cell migration and increase in apoptosis. **(C)** syngeneic mice model with flank tumor (mEER cell line injection) followed by treatment with CBD which enhances the infiltration of immune cells in the Tumor Immune Micro-Environment (TIME). Created in https://BioRender.com.

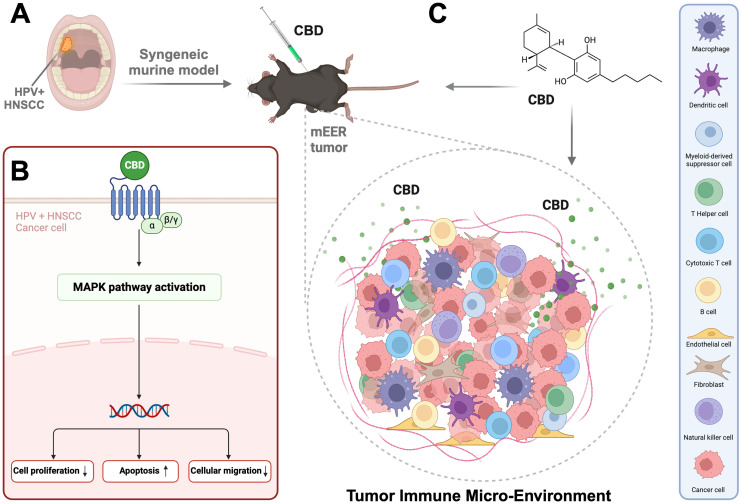

## 1 Introduction

Head and neck squamous cell carcinoma (HNSCC) is the sixth most common malignancy worldwide and affects more than 69,000 people in the US annually (1). The two major types of HNSCC include human papillomavirus-mediated (HPV-positive) HNSCC, and HPV-negative HNSCC which is primarily caused by consumption of tobacco, smoking, and alcohol (2). HPV-positive HNSCC accounts for roughly 4% of all cancers worldwide with a projected 30,000 cases in 2029 in the USA (3). Over the past few decades, the US has seen a dramatic increase (>5-fold higher) in daily use of recreational cannabinoids, with more varied routes of consumption such as ingestion and vaping, and an increase in the purity of specific cannabinoids, including tetrahydrocannabinol (THC) and cannabidiol (CBD) (4, 5), which is a component of marijuana plant (*Cannabis sativa L)*. In the US, during the year 2022, the usage of marijuana reached its highest (28%) among the age group of 35–50 years (6). Among these components, THC has addictive properties and causes psychotropic behaviors among consumers whereas CBD is reported to have relatively reduced psychotropic effects compared to THC (7). Use of CBD in cancer patients undergoing active cancer therapy has also increased to 20-30% recently (8, 9).

The endocannabinoid system is regulated by the Cannabinoid-specific G protein-coupled receptors (GPCRs) Cannabinoid receptor 1 (CB1), which is abundantly found in the central nervous system (CNS), and Cannabinoid receptor 2 (CB2), which is highly expressed in B cells and natural killer cells and other GPCRs that have different physiologic roles (10). Activation of CB1 and CB2 via different immunologic and tumor-intrinsic pathways has led to tumor promotion in colorectal carcinoma (11, 12), hepatocellular carcinoma (13), glioblastoma, lung carcinoma (14), esophageal cancer (15), prostate cancer (16) pancreatic cancer (17) ovarian cancer (18), breast cancer (19) and others (20–23). We previously observed that selective activation of CB1 and CB2 as well as non-selective activation of cannabinoid receptors by THC in cell lines and animal models could promote cell growth and migration, and inhibit apoptosis through MAPK pathway in HPV-positive HNSCC (24). In contrast, several studies suggest an inhibitory activity of CBD in a variety of solid tumor systems. In triple-negative breast cancer models, CBD significantly inhibits epidermal growth factor (EGF)-induced proliferation, chemotaxis, and activation of ERK and AKT signaling pathway (25). CBD enhances the susceptibility of lung cancer cells to adhere to and subsequently be lysed by lymphocyte-activated killer cells and synergizes with bortezomib in the inhibition of multiple myeloma cells (26, 27).

In addition, cannabinoids are also known to influence the immune system including the expressions of cytokines, T cells, and stabilize the activities of synaptic inflammation and degeneration (28–30). Several reports suggested that suppression of inflammatory sites might be correlated to cannabinoid receptors which are arbitrated by CBD (31). In another study, it was observed that administration of CBD suppressed cytokine production in both normal and double CBD receptor knockout mice (Cnr1^-/-^/Cnr2^-/-^) (32). Nevertheless, there are context-dependent targets of CBD that show its immunomodulatory properties offering the fact that it can be used as a therapeutic agent to cure inflammatory diseases (28). Recent studies suggested the anti-cancer properties of CBD in HNSCC cells as well as in an immunocompromised murine xenograft model (33). However, these studies did not explore the role of CBD in modulating tumor immune microenvironment by using syngeneic mice models. The impact of CBD ligands on the immune system is not well understood and the physiologic and immunological role of CBD in HNSCC is yet to be explored. Hence, in this study, we investigated the impact of CBD in HPV-positive HNSCC using a series of *in vitro* and *in vivo* models and explored its interaction with the immune microenvironment by using syngeneic HPV-positive mouse models.

## 2 Materials and methods

### 2.1 Cell culture

The human HPV positive HNSCC cell line UPCI: SCC154 was purchased from American Type Culture Collection (#CRL-3241, ATCC) and UD-SCC-2 (RRID: CVCL_E325) was obtained from the Dr. Silvio Gutkind’s Laboratory at the Moores Cancer Center, University of California San Diego. UPCI: SCC154 is a HPV positive HNSCC cell line derived from a squamous cell carcinoma of the tongue of a 54 year old white male. UD-SCC-2 is also a HPV positive HNSCC cell line derived from squamous cell carcinoma of the hypopharynx of a 58 year old male. These cell lines are well characterized HPV positive HNSCC cell lines (34–37). The cells were cultured in Dulbecco’s modified Eagle’s medium (DMEM) supplemented with 10% fetal bovine serum (FBS) plus penicillin (50 U/ml) and streptomycin (50 μg/ml). The mouse tonsil epithelial cells expressing HPV-16 E6 and E7 and H-Ras (mEER) (38–40) were cultured in E-Media containing 67% DMEM (Fisher Scientific), 10% FBS (Invitrogen), 22% Ham’s F-12 Nutrient Mix (Fisher Scientific), 1% penicillin/streptomycin (Invitrogen), 25µg/ml hydrocortisone (Sigma), 5µg/ml insulin (Sigma), 5µg/ml transferrin (Sigma), 1.36ng/ml tri-iodo-thyronine (Sigma), 8.4ng/ml cholera toxin (Sigma) and 5ng/ml epidermal growth factor (Invitrogen). The cells were maintained at 37°C under an atmosphere of 5% CO_2_.

### 2.2 Cell proliferation assay

The proliferation of HPV-positive HNSCC cells UD-SCC-2 and UPCI: SCC154 upon treatment with Cannabidiol (CBD) (Cayman Chemicals) was determined by using AquaBluer Solution (MultiTarget Pharmaceuticals). Briefly, 1 x 10^5^ cells/well were seeded overnight into 96-well plates and the next day treated with 10μM of CBD and Vehicle (1:9 DMSO: PBS) as the control for 24 and 48 hours. The cells were stained with 1X AquaBluer Solution for 3 hours at 37°C and fluorescence intensity was measured at 540nm for excitation and 580nm for emission by using a microplate reader (TECAN Spark).

Furthermore, the incorporation of 5-bromo-2’-deoxyuridine (BrdU) into cellular DNA during cell proliferation was measured using an anti-BrdU antibody in HPV-positive HNSCC cells UD-SCC-2 and UPCI: SCC154. Cells were seeded at a density of 1 × 10^5^ cells/well into 96-well microtiter plates overnight followed by treatment with 10μM of CBD for 24 and 48 hours. After incubation with drugs, the cells were incubated with 1X BrdU for 24 h. The BrdU assay kit was purchased from Cell Signaling Technology (Danvers, MA, USA). BrdU assay was carried out as described previously by Sen et al. (41). The intensity was measured at 450 nm using a microplate reader (TECAN Spark). The percentage of cell proliferation was calculated as relative to that of the control values using GraphPad Prism 10.4.0 software.

### 2.3 Apoptosis assay

The HPV-positive HNSCC cell lines UPCI: SCC154 and UD-SCC-2 were used to determine apoptosis using APC Annexin V Apoptosis Detection Kit with PI (BioLegend, San Diego, CA, USA) after treatment with 10μM of CBD. Briefly, 1×10^5^ cells/well were plated into 12-well plates followed by treatment with 10μM of CBD for 24h and 48h. Cells were stained according to the manufacturer’s instructions and were analyzed using the NovoCyte Flow Cytometer Systems (Agilent Technologies). Data analysis was conducted using FlowJo (FlowJo, LLC).

### 2.4 Wound healing assay

A scratch or wound healing assay was performed to study cellular migration after treatment with 10μM of CBD in HPV-positive cell lines UD-SCC-2 and UPCI: SCC154 as reported earlier (41). The images of the wound were captured at 0h, 12h and 24 h using an inverted microscope (ECLIPSE Ts2R, Nikon) at 4X magnification. The images were analyzed using Image J software ver. 1.54 (NIH, Bethesda, MD, USA). The percentage of cell migration was calculated relative to the control using GraphPad Prism 10.4.0 software.

### 2.5 Western blot analysis

Western Blot was carried out for HPV-positive HNSCC cells UD-SCC-2 and UPCI: SCC154, post-treatment with CBD 1μM, 5μM, and 10μM for 15 and 30 minutes as reported earlier (24). The primary antibodies p38 (#8690), phospho- p38 (pp38) (#4511), Erk1/2 (#4370), phospho-extracellular signal-regulated kinase (pERK1/2), (#4695), SAPK/JNK (#9252), phospho-c-Jun N-terminal kinase (pJNK) (#4688), MAPKAPK-2 (#3042) and phospho-mitogen-activated-protein-kinase-activated-protein-kinase-2 (pMK-2) (#3007) and Vinculin (#4650) were obtained from Cell Signaling Technology (Danvers, MA, USA) and used at a dilution of 1:1000. Anti-rabbit IgG, HRP-linked Antibody (#7074, Cell Signaling Technology (Danvers, MA, USA)) was used as the secondary antibody at a dilution of 1:2000. The blots were developed using Immobilon Western Chemiluminescent HRP Substrate (Millipore Sigma, Burlington, MA, USA) and images were captured by iBright FL1500 imaging system (Thermo Fisher Scientific, Waltham, MA, USA). Vinculin was used as loading control. The western blot images were analyzed using the Image J software ver. 1.54 (NIH, Bethesda, MD, USA). The relative protein expression was measured with respect to control after normalizing with loading control (Vinculin). The ratios for pp38:p38, pERK1/2:ERK1/2, pSAPK: SAPK and pMK2:MK2 were measured after normalizing the band intensity of the respective protein markers with Vinculin. The original blots can be found in **Supplementary Figures S2A-D**.

### 2.6 Syngeneic murine models

Six weeks old male mice (wild type C57BL/6J mice, athymic nude mice and B6.129S7-Rag1^tm1Mom^/J or Rag1 -/- also commonly known as Rag1 Knockout (Rag 1KO)) mice were obtained from The Jackson Laboratory (Bar Harbor, ME, USA). HPV-positive mEER cells, which are immortalized with a retrovirus containing HPV16 E6, HPV16 E7, and H-Ras genes (39, 40, 42), were used at a concentration of 1x10^6^ cells/mouse in 100μl of 1X PBS to develop subcutaneous tumors in the in the flank region of wild-type C57BL/6J mice, athymic nude mice which are immunodeficient, and Rag1 KO mice which do not produce mature T cells or B cells. Palpable tumors in the flank were observed 5 days after injection of cells and the mice were divided randomly into two groups. Next day (Day 6), the wild-type C57BL/6J mice, the athymic nude mice and Rag1 KO mice were treated with 10μM CBD and control vehicle (1:9 DMSO: PBS) intraperitoneally (I.P) every day and continued till Day 21. In addition, for CD4+T cells and CD8+T cells depletion in wild-type C57BL/6J mice, 1x10^6^ mEER cells/mouse was subcutaneously injected in the flank region of each of the mice. Mice were injected with 200 µg of anti-CD4 (#BE0003-3, Bio X Cell, Lebanon, NH, USA), 200 µg of anti-CD8 (#BE0061, Bio X Cell, Lebanon, NH, USA) and 200 µg of IgG (#BE0093, Bio X Cell, Lebanon, NH, USA) intraperitoneally 2 days prior to the injection of mEER cells, 2 days after and finally on the 7th day after the injection of mEER cells in mice. In addition, the mice were treated with or without 10μM of CBD every day and continued till Day 21. Tumors were measured with an external caliper, and the tumor volume was calculated as: (length x width^2^)/2. All experimental protocols were approved by the Institutional Animal Care and Use Committee of UCSD (#S16200) and maintained under specific pathogen-free conditions at Moores Cancer Center with the approval of the Institutional Animal Care and Use Committee of the University of California San Diego.

### 2.7 Immuno-flow cytometry

Flank tumors after treatment with CBD and vehicle from wildtype mice C57BL/6J mice were harvested on Day 15, minced, and resuspended by using Tumor Dissociation Kit (Miltenyi Biotec) diluted into DMEM for subsequent processing with the gentleMACS Octo Dissociator, according to the manufacturer’s recommendations for tumor dissociation into a single-cell suspension. Digested tissues were then passed through 70 µm strainers to produce a single-cell suspension. Samples were washed with PBS and processed for live/dead cell discrimination using Zombie viability stains (BioLegend). Cell suspensions were then washed with cell staining buffer/FACs Buffer (BioLegend) prior to cell surface staining, performed at the indicated antibody dilutions for 30 min at 4 °C, and protected from light. Cell surface staining was performed for 30 min at 4 °C with the following mouse antibodies from BioLegend: CD45 (#30-F11, 1:100), CD3 (#17A2, 1:100), CD8 (#53-6.7, 1:100), CD4 (#RM4-4, 1:100), CD19 (#6D5, 1:100), NK1.1 (#PK13, 1:100), Ly6C (#HK1.4, 1:100), CD11b (#M1/70, 1:100), CD11c (#N418, 1:100), MHCII (#M5/114.15.2, 1:100), Ly6G (#1A8, 1:100) and XCR1 (#ZET, 1:100). Stained cells were washed and then fixed with BD cytofix (#554655, BD Biosciences, New Jersey, USA) for 20 min at 4 °C, protected from light. Finally, cells were washed and resuspended in FACS buffer and acquired using the BD LSRII Fortessa. Downstream analysis was performed using FlowJo, (version 10.6.2). Absolute T-cell numbers per milligram of tumor were derived from CD45+ -cell counts in 100μL cell suspension divided by tumor weight.

### 2.8 Immunohistochemistry

Flank tumor tissues from wildtype mice C57BL/6J mice were collected and paraffin-fixed at the Day 22. Tissue blocks were prepared and processed for IHC analysis. The IHC analysis was performed for CD4+T cells, CD8+T cells and CD19+B cells as reported earlier (43). The Images were analyzed using the QuPath Software version 0.4.3.

### 2.9 Multiplex IHC

The 5μm paraffin sections from flank tumor tissues from wildtype C57BL/6J mice were subjected for multiplex IHC analysis. First, the sections were baked at 60°C for 1 hour followed by tissue rehydration through successive steps of xylene, 100% ethanol, 95% ethanol, 70% ethanol and finally rinsing with distilled water. After rinsing the slides antigen retrieval was done by incubating them in a citrate based (pH 6.0) antigen unmasking solution (Vector Laboratories, Newark, CA, USA) at 95°C for 30 minutes. Slides were then cooled down to room temperature and were blocked using Peroxidase blocking, Bloxall Solution (Vector Laboratories, Newark, CA, USA) for 10 minutes. The slides were then washed with 1X TBST, followed by incubating with primary antibody anti-CD8 (Invitrogen, Waltham, MA, USA) for 1 hour, followed by washing with 1X TBST. Next, the slides were incubated with anti-Rat HRP Polymer secondary antibody (Cell IDX, San Diego, CA, USA) for 30 minutes followed by washing. Finally, the sections were incubated with fluorophore Opal 570 (Akoya Biosciences, Marlborough, MA, USA) for 10 minutes. The same incubation steps were repeated for the other primary antibodies such as anti-CD4 (Abcam, Cambridge, UK), anti-pp38 (Cell Signaling Technology, Danvers, MA, USA), anti-PanCK (Dako, Glostrup, Denmark) and anti-CD45 (Abcam, Cambridge, UK). The slides were washed with 1X TBST and incubated with secondary antibody anti-Rabbit HRP Polymer for 30 minutes. Slides were again washed with 1X TBST and incubated with fluorophore Opal 690, Opal 620, Opal 520 and Opal 780 (Akoya Biosciences, Marlborough, MA, USA) for 10 minutes respectively. After the final incubation with fluorophores, slides were washed with 1X TBST, followed by washing with distilled water and then incubation with DAPI (1µg/ml) for 15 minutes. Finally, slides were mounted on a coverslip with Vectashield Vibrance (Vector Laboratories, Newark, CA, USA). Images were analyzed for colocalization of CD4, CD8 and pp38 using the QuPath Software version 0.4.3 and protocol created by Lumanto et al. (44).

### 2.10 Statistical analysis

Statistical analysis was performed for three independent experiments by using GraphPad Prism 10.4.0 software.

Student’s t-test was performed to assess the statistical significance. P<0.05 or less was considered for statistical significance.

## 3 Results

### 3.1 CBD treatment inhibits cell proliferation and migration of HPV-positive HNSCC cells by promoting apoptosis

**Figure 1:**
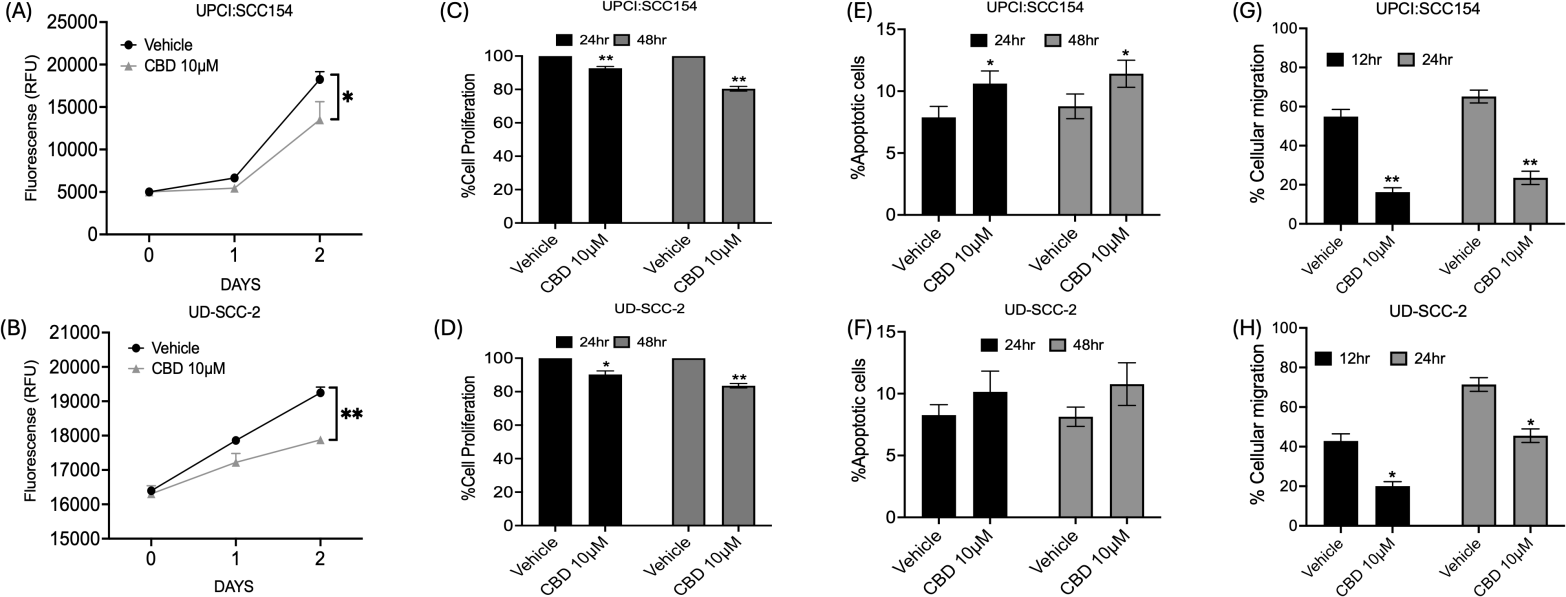
CBD treatment promotes anti-cancer activity in HPV-POSITIVE HNSCC cells. Measurement of cell proliferation by AquaBluer Assay after treatment with 10 μM of CBD in HPV-positive HNSCC cells **(A)** UPCI: SCC154 and **(B)** UD-SCC-2 for 24 and 48 hours. Graphical representations of percentage of cell proliferation by BrdU assay on **(C)** UPCI: SCC154 and **(D)** UD-SCC-2 after treatment with CBD 10 μM for a time period of 24 h and 48h. Cell proliferation decreases significantly with CBD treatment compared to Vehicle treated group. Measurement of percentage of apoptosis post CBD treatment for 48 hours in HPV-positive HNSCC cells **(E)** UPCI: SCC154 and **(F)** UD-SCC-2. Measurement of percentage of cellular migration post CBD treatment for 48 hours in HPV-positive cells **(G)** UPCI: SCC154 and **(H)** UD-SCC-2. Statistical analysis was performed by unpaired Student’s t-test. [*p<0.05 **p<0.01].

To determine the nature of CBD-mediated inhibition of tumor growth, cell proliferation was assessed in HPV-positive HNSCC by AquaBluer solution as well as a BrdU assay. It was observed that 10 μM of CBD treatment significantly decreased cell proliferation of HPV-positive HNSCC cells as compared to vehicle treated cells (**Figures 1A-D**). Next, we evaluated the ability of CBD to induce apoptosis in HPV-positive HNSCC cancer cells following treatment with 10 μM of CBD for 24 and 48 hours by Annexin-V/PI staining. A significant increase in the percentage of apoptotic cells upon treatment with 10 μM of CBD was observed as compared to the vehicle counterpart for UPCI: SCC154 cells (**Figure 1E**). In UD-SCC-2 cells, although we observed an increase in the percentage of apoptosis after treatment with 10 μM of CBD, but it was not statistically significant (**Figure 1F**). Enhanced cellular migration is a phenotypic characteristic of malignancy (45), thus, in this study, we interrogated the ability of CBD in inhibiting cellular migration by using the wound healing or scratch assay. The percentage of cellular migration of HPV-positive cell lines UD-SCC-2 and UPCI: SCC154 was determined after treatment with a vehicle and 10 μM of CBD for 12 and 24 hours. Wound healing assay showed CBD treatment significantly decreased the percentage of cellular migration as compared to the vehicle after 12 hours and 24 hours in HPV-positive HNSCC cells (**Figures 1G, H**). These results collectively demonstrate the anti-cancer properties of CBD in HPV-positive HNSCC cells by inhibiting proliferation and migration of HPV-positive HNSCC cells and by promoting apoptosis using *in vitro* assays.

### 3.2 CBD activates the MAPK pathway in HPV-positive HNSCC cells

**Figure 2:**
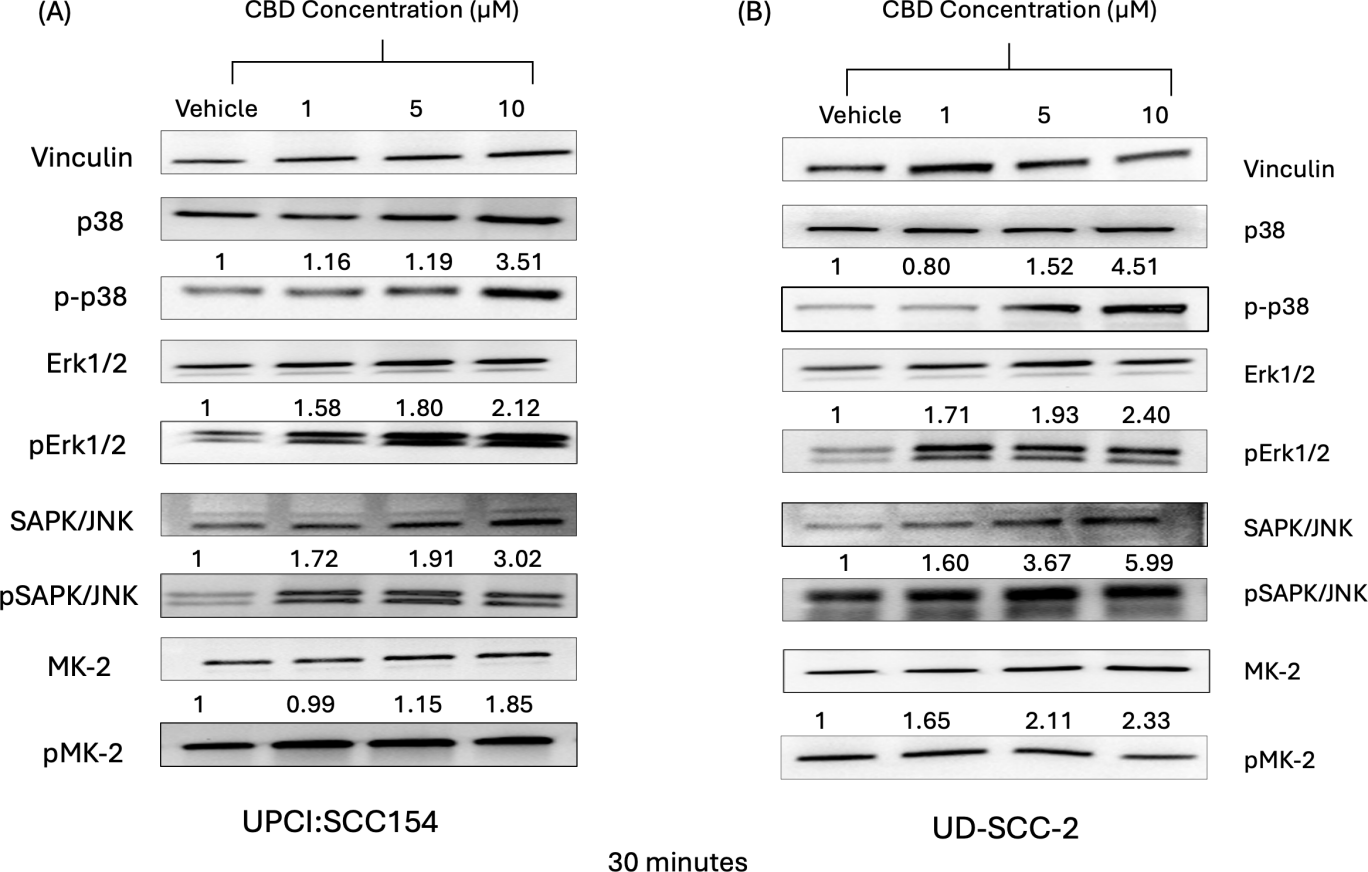
CBD treatment promote activation of markers of MAPK pathway in HPV-positive HNSCC cells. Western blot analysis of MAPK pathway markers like p38, ERK1/2, JNK/SAPK and MK2 and it’s signaling after treatment with 10 μM of CBD in HPV-positive HNSCC cells **(A)** UPCI: SCC154 and **(B)** UD-SCC-2 for 30 minutes.

It is well known that the mitogen-activating protein kinase (MAPK) pathway plays an essential role in cell proliferation, differentiation, and senescence (46). In a previous study by our group, we observed that stimulation of cannabinoid receptors by its agonists promotes the activation of p38 MAPK pathway (24). Previous reports suggest activation of MAPK pathway upon CBD stimulation promotes apoptosis (47, 48). In accordance with the previous studies, we also observed that CBD treatment (1-10 µM) increased the expression of MAPK markers such as pp38, pERK1/2, pJNK and pMK-2 in a dose-dependent manner after 15 minutes (**Supplementary Figures S1A, B**) and 30 minutes (**Figures 2A, B**) compared to vehicle treated HPV-positive HNSCC cells. These results suggested that stimulation with CBD led to the activation of MAPK pathway along with its other markers such as ERK1/2, JNK/SAPK and MK2 in HPV-positive HNSCC cells.

### 3.3 CBD treatment promotes anti-tumor activity in syngeneic mouse models of HNSCC

**Figure 3:**
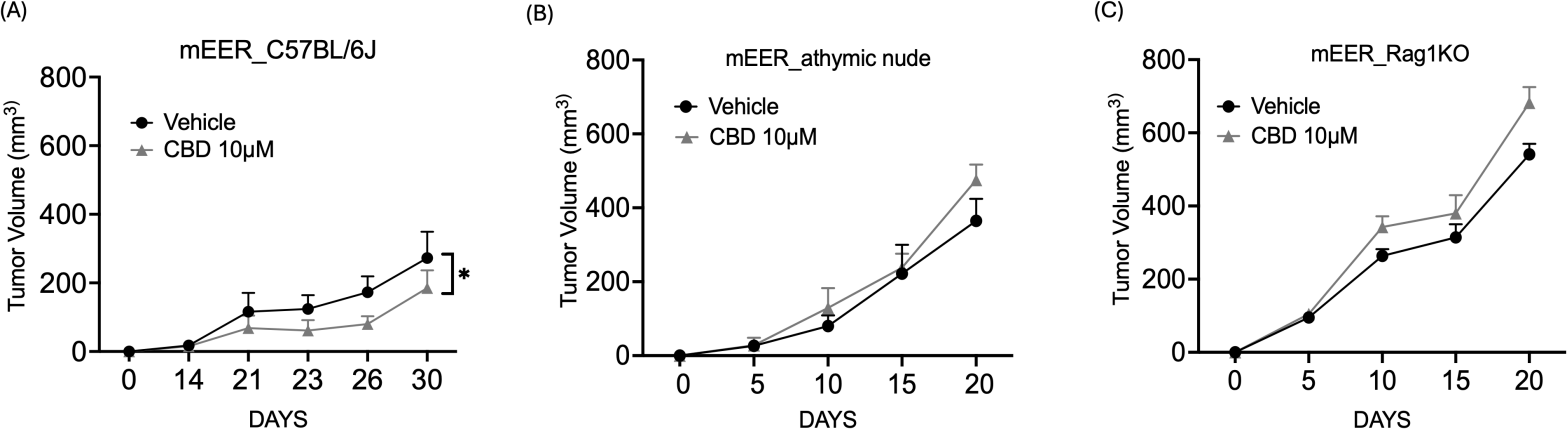
CBD treatment promotes anti-tumor activity in syngeneic mouse models of HNSCC. Tumor volume of mice injected with 1x10^6^ mEER cells/mouse in the flank region of **(A)** immunocompetent syngeneic C57BL/6J mice model (wild-type), **(B)** athymic nude mice and **(C)** Rag1 KO. From Day 6, the mice were treated with 10 μM of CBD and vehicle control (1:9 DMSO: PBS) intraperitoneally for everyday and continued till Day 21. Statistical analysis was performed by unpaired Student’s t-test [*p<0.05].

Next, to evaluate the anti-tumor activity of CBD *in vivo*, both immunocompetent and immunocompromised syngeneic mice models (wild-type C57BL/6J mice, athymic nude mice, and Rag1 KO mice) were used. In the immunocompetent syngeneic C57BL/6J mice model (wild-type), we observed that treatment with 10 μM of CBD significantly decreased the tumor volume as compared to the vehicle-treated group on Day 21 (**Figure 3A**). In contrast, no significant difference in tumor volume was observed between vehicle and 10 µM of CBD treated athymic nude mice and Rag1 KO mice on Day 21 (**Figures 3B, C**). These results suggested that the effect of CBD on tumors might be immune-modulated.

**Figure 4:**
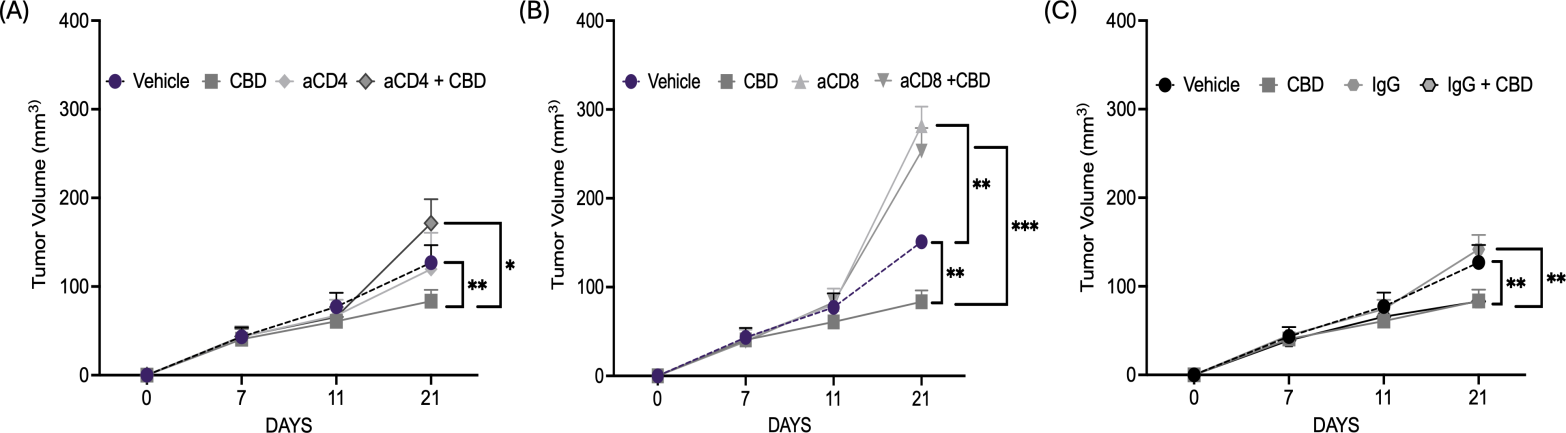
CBD treatment facilitates CD4+T and CD8+ T cells mediated anti-tumor immune response in syngeneic mouse models of HNSCC. **(A)** Tumor volume of mice injected with 1x10^6^ mEER cells/mouse in the flank region of C57BL/6J mice model (wild-type). The mice were treated with vehicle control, 10 µM of CBD, anti-CD4 and its combination intraperitoneally for everyday and continued till Day 21. **(B)**. Tumor volume of mice injected with 1x10^6^ mEER cells/mouse in the flank region of C57BL/6J mice model (wild-type) and treated with vehicle control, 10 µM of CBD, anti-CD8 and its combination. **(C)** Tumor volume of mice treated with IgG antibody in the presence and absence of 10 µM of CBD. Statistical analysis was performed by unpaired Student’s t-test [*p<0.05 **p<0.01, ***p<0.001].

Next, to further evaluate the immune-modulatory activity of CBD, CD4+T and CD8+ T cell depletion was performed in immunocompetent C57BL/6J wild-type mEER mice model. As observed earlier, the mice treated with 10 µM of CBD showed a significant decrease in tumor volume at the end of Day 21 compared to vehicle-treated controls. In contrast, depletion of CD4+T cells resulted in an increase in tumor volume and CBD treatment enhanced the tumor growth in CD4-depleted mice suggesting tumor growth-promoting effects of CBD in the absence of CD4+T cells and a key role of CD4+T cells in CBD-mediated anti-tumor activity (**Figure 4A**). Next, we observed that depletion of CD8+T cells resulted in an increase in tumor volume, however, no significant difference in tumor volume was observed in CD8-depleted mice after CBD treatment (**Figure 4B**). However, mice treated with IgG antibody with or without treatment with 10 µM of CBD exhibit similar results as observed in mice treated with or without CBD (**Figure 4C**). Taken together, these results suggested that CBD-mediated tumor inhibition is mediated by both CD4+T and CD8+T cells, and that CBD can be growth stimulatory *in vivo* in the absence of CD4+T cells, suggesting a powerful influence of CD4+T cells in mediating CBD immune inhibition of tumor growth.

### 3.4 CBD treatment modulates infiltration of immune cells in the tumor immune micro-environment

**Figure 5:**
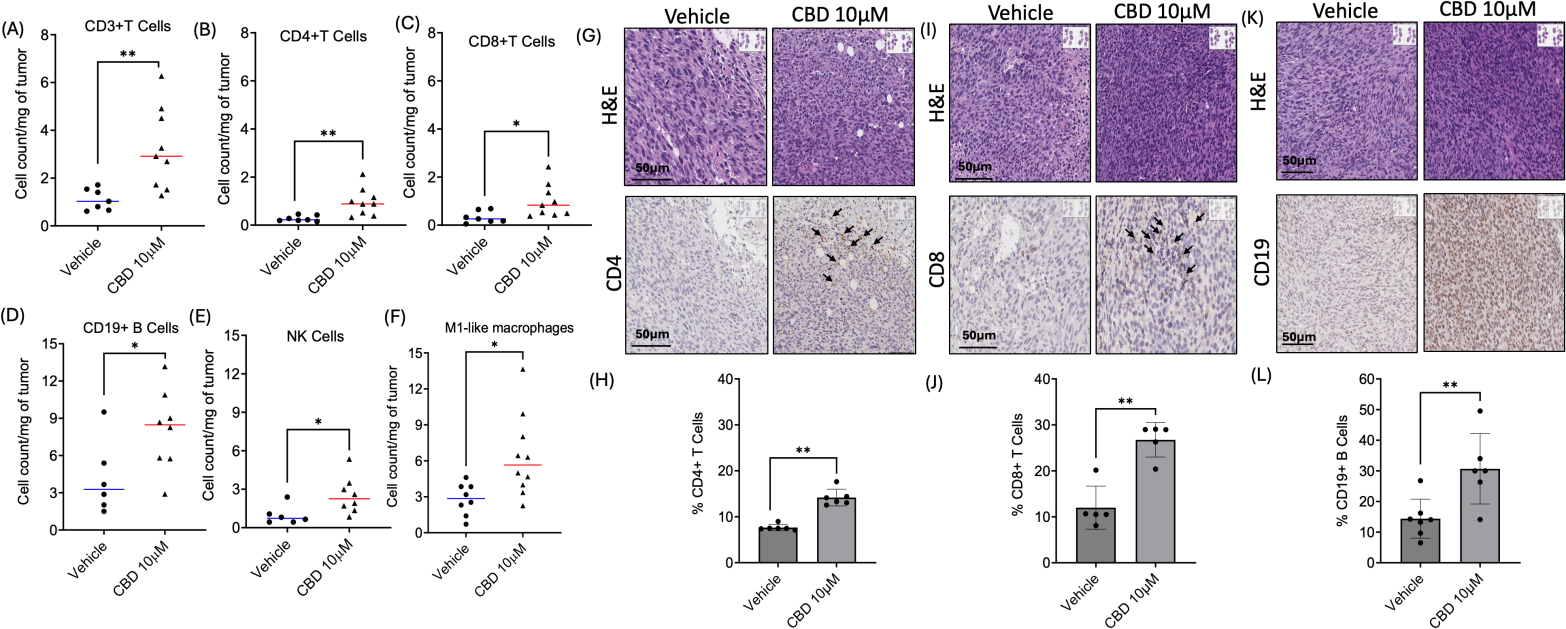
CBD treatment modulates infiltration of immune cells in the Tumor Immune Micro-Environment (TIME). Identification of immune cells infiltration in 10 µM of CBD treated wild-type mEER tumors by flow cytometric analysis for **(A-C)** T cell markers (CD3+, CD4+, and CD8+T), **(D)** B cells marker (CD19+), **(E)** natural killer (NK) cells, and **(F)** M1-like macrophages compared to the vehicle treated mice tumors at the end of Day 15. Immunohistochemical (IHC) staining of **(G)** CD4, **(I)** CD8 and **(K)** CD19 in tumor sections of CBD and vehicle treated mice at the end of Day 21. Graphical representation for **(H)** % CD4+T cells, **(J)** % CD8+T cells and **(L)** CD19+B cells. Statistical analysis was performed by unpaired Student’s t-test [*p<0.05 **p<0.01].

Next, we explored the role of CBD in modulating infiltration of immune cells in TIME, by immune-flow cytometry analysis on flank tumors of C57BL/6J wild-type mice treated with or without 10 μM of CBD (**Supplementary Figure S3A**). Tumors were harvested on Day 15 and single-cell suspensions were acquired from the harvested tumors and the cell surface was stained with the following mouse antibodies: immune cells (CD45+), T lymphocytes (CD3+, CD8+, and CD4+), B lymphocytes (CD19+), natural killer cells (CD3-NK1.1+), conventional Type 1 dendritic cells (cDC1) (CD11b-CD11c+MHCII+XCR1+), monocytic myeloid-derived suppressor cells (M-MDSCs) (CD11b+MHCII-Ly6CHighLy6G-) and polymorphonuclear MDSCs (PMN-MDSCs) (CD11b+MHCII-Ly6ClowLy6G+), macrophages (M1-like/anti-tumor: CD11b+Ly6C- MHCII+ and M2-like/pro-tumor: CD11b+Ly6C-MHCIIint). Flow cytometric analysis revealed a significant increase in the absolute count per milligram of the tumor tissue for CD3+ (**Figure 5A**), CD4+ (**Figure 5B**), and CD8+ (**Figure 5C**) T cells along with CD19+ B cells (**Figure 5D**), natural killer cells (**Figure 5E**), and M1-like macrophages (**Figure 5F**) in the CBD-treated mice as compared to the vehicle-treated mice. On the contrary, there was no significant difference for cDC1s, M-MDSCs, PMN-MDSCs, and M2-like macrophages in the CBD-treated mice group compared to the vehicle-treated mice (**Supplementary Figures S3B-E**).

Next, we validated the infiltration of CD4+ T cells, CD8+ T cells, and CD19+ B cells upon CBD stimulation by IHC analysis in tumor sections obtained from immune-competent C57BL/6J wild-type mEER mice. The tumors were harvested after Day 21 for flank tumors upon completion of the treatment (vehicle and 10 μM of CBD). IHC results indicated a significant increase in the percentage of CD4+T cells (**Figures 5G, H**), CD8+T cells (**Figures 5I, J**), and CD19+B cells (**Figures 5K, L**) in the tumor sections of 10 μM CBD-treated mice as compared to the vehicle counterparts.

### 3.5 CBD treatment promotes colocalization of CD4+T and CD8+T cells along with phospho-p38 in the tumor microenvironment of syngeneic mouse model

**Figure 6:**
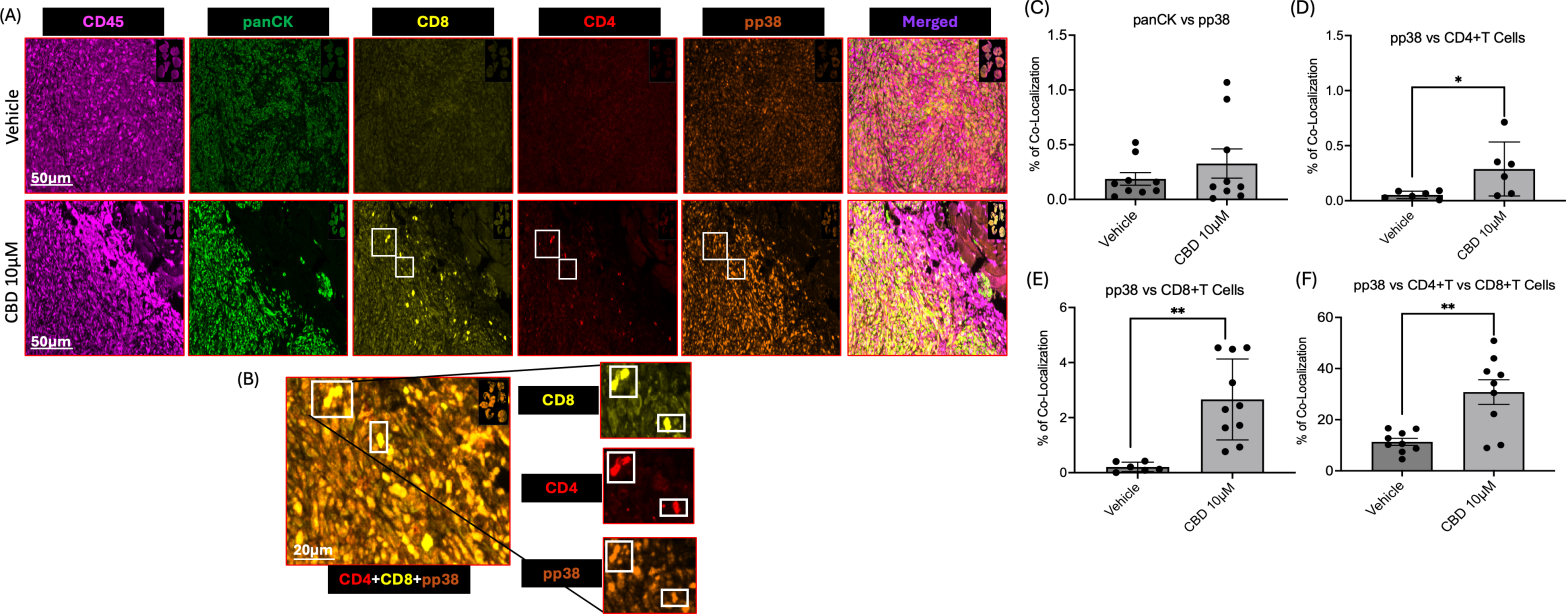
CBD treatment promotes colocalization of CD4+T and CD8+T cells along with phospho-p38 (pp38) in the tumor microenvironment of a syngeneic mouse model. **(A)** Representative images of multiplex IHC analysis to evaluate the staining of CD45, panCK, CD4, CD8 and pp38 MAPK in mice tumor sections obtained from vehicle and CBD-treated groups. **(B)** Representative images of multiplex IHC analysis to show colocalization of CD4+T cells, CD8+T cells along with pp38 MAPK for the tumor sections obtained from CBD-treated mice group. **(C-F)** Graphical representation of % of co-localization of panCK:pp38, pp38:CD4+T Cells, pp38:CD8+T Cells and pp38:CD4+T and CD8+T Cells respectively. Statistical analysis was performed by unpaired Student’s t-test [*p<0.05 **p<0.01].

Furthermore, we performed multiplex IHC to evaluate the infiltration of CD4+T cells and CD8+T cells along with activation of MAPK pathway in mice tumor sections obtained from vehicle and CBD-treated groups. We observed that increased accumulation of immune cell markers such as CD45, CD4+T cells, and CD8+T cells in CBD-treated mice tumor sections as compared to vehicle-treated counterparts. In accordance with our *in vitro* study, we also observed activation of MAPK pathway in CBD-treated mice tumor sections as evident from increased phosphorylation of p38 (**Figures 6A, B**). Along with these findings we also observed no significant difference between vehicle and CBD treated tumors for co-localization of panCK with pp38 (**Figure 6C**). On the contrary co-localization of CD4+T cells and CD8+T cells with pp38 were significantly higher in the CBD treated group compared to the vehicle treated group (**Figures 6D-F**). These results suggested the role of CBD in promoting colocalization of CD4+T cells and CD8+T cells with concurrent activation of pp38 MAPK which is related to cellular proliferation, apoptosis, differentiation, development, and inflammatory responses (47). Thus, CBD in the tumor microenvironment may promote direct activation of CD4+T cells and CD8+T cells and their interaction between immune cells and tumor cells.

## 4 Discussion

HNSCC mainly arises from the oropharynx region which includes the base of tongue, tonsil, and oropharyngeal walls and it is increasing in the US with 70% accredited to HPV-positive HNSCC (49). Multiple case-control studies showed a positive association of marijuana with oropharyngeal cancers and a recent large population-based study showed an association of HNSCC with marijuana use disorder (50). In this study, we investigated the anti-cancer properties of CBD and its role in immune modulation by using a series of *in vitro* and *in vivo* HPV-positive HNSCC models. Prior studies provide data regarding modulation of the endocannabinoid system for tumor inhibition or proliferation. The cannabinoid concentration used for tumor inhibition via CB1 and CB2 agonists ranges between 5-20 µM. It has been observed that the THC concentration found in the blood and plasma of marijuana smokers rarely exceeds 1 µM and for CBD the range is from 1-10 µM with recreational exposure, so we purposefully chose dosages found within normal ranges found during common exposures (51–55). A recent study by Go et al. reported that in HNSCC cells, CBD promotes apoptosis in a dose-dependent manner (33). Likewise, in this study we observed that HPV-positive HNSCC cell lines (UPCI: SCC154 and UD-SCC-2) treated with 10 µM of CBD for 24 hours and 48 hours exhibit increased the percentage of apoptotic cells, inhibited cell proliferation and migration compared to vehicle-treated cells. These findings were in line with previous studies in breast, colorectal, leukemia, and pancreatic tumor cells which further validates the fact that CBD can induce anti-tumor properties in *in vitro* solid tumor systems (56–59).

It is well known that the mitogen-activating protein kinase (MAPK) pathway has been implicated in regulation of cell cycle arrest, apoptosis, senescence and differentiation (60). It can exhibit a dual role in cancer progression and suppression in context dependent manner. Several studies suggested that p38 MPAK induces cell cycle arrest by promoting apoptosis and thereby inhibit cell proliferation (61, 62). In contrast, many studies reported activation of p38 MAPK promote proliferation of cancer cells and further contribute towards metastasis (63). Dysregulation of this pathway has been demonstrated in a variety of malignancies, including hepatocellular carcinoma, gastric adenocarcinoma, and renal cell carcinoma (64–67). In our previous study led by Chao et al., we demonstrated that THC, a component of marijuana exhibits activation of cannabinoid-specific G protein-coupled receptors (GPCR), CNR1 (Cannabinoid Receptor 1), and CNR2 (Cannabinoid Receptor 2) and drives proliferation and invasion of HPV+ HNSCC via the activation of the mitogen-activated protein kinase (MAPK) pathway (24). In contrast, in this study, we observed that in HPV-positive HNSCC cells CBD treatment exhibit anti-cancerous properties by promoting activation of p38 MAPK pathway and other MAPK pathways such as ERK and JNK. These results suggested the differential role of THC and CBD where THC exhibit oncogenic activity and CBD promote anti-cancer properties. It is well known that THC and CBD also exhibit different receptor specificity (68) which may reinforce the context dependent activation of p38 MAPK pathway.

Although the immune network can be modified depending on its activation status and stimulus by cannabinoids, the immunological role and its mechanism of action of CBD in the tumor immune microenvironment of HNSCC is still not clear (28). A study by García-Morales et al. showed that CBD was able to reduce the advancement of breast cancer tumors *in vivo* (69). In this study, we investigated the role of CBD in immune modulation in a tumor microenvironment by using a series of *in vivo* models of HNSCC. Our results suggested that in these *in vivo* models of HPV-positive HNSCC, CBD promotes anti-tumor activity by modulating the immune system. It is well known that T lymphocytes play an important role in promoting anti-tumor immune response. CD4+T cells bind with the major histocompatibility complex (MHC) class II and help increase cytokine production by T helper cells (70) and the CD8+T cells interact with MHC class I molecules to show their immune defense towards pathogens, viruses, and bacteria, and also help the immune system to recognize cancerous cells and eliminate them eventually (71). This led us to further investigate the role of CD4+T and CD8+T cells in the presence and absence of CBD in the immune system of the wild-type murine model. We observed that the absence of CD4+T and CD8+ T cells promotes tumor growth compared to vehicle-treated group. However, we observed a significant increase in tumor volume in CD4-depleted mice treated with CBD as compared to CBD-treated wild-type mice suggesting the importance of CD4+T cells in CBD-mediated anti-tumor activity. These findings were in accordance with a previous study by Cosentino et al. where authors found that CBD can modulate CD4+ T cell differentiation and cytokine mRNA expression in human PBMCs (72, 73). However, no significant difference in tumor volume was observed in CD8-depleted mice with or without CBD treatment in our study.

The presence of immune cells, primarily T-lymphocytes, dendritic cells, B cells, plasma cells, some natural killer cells, macrophages, and eosinophils are associated with tumor initiation and progression of HNSCC (74). However, HNSCC is a tumor with a dominant immune suppressive microenvironment (75, 76). In this study, we observed that CBD exerts an anti-tumor activity in immunocompetent mouse models, which further prompted us to explore the specific immune cell population responsible for CBD-mediated immune modulation. Based on the flow cytometry, a significant increase in absolute count of CD3, CD4+T cells, CD8+T cells along with CD19+B cells, natural killer cells, and M1 macrophages per mg of tumor was observed in the CBD-treated tumor tissues compared to the vehicle-treated group. These results are in line with a previous study where the authors observed the presence of immune cells, T-lymphocytes, B cells and plasma cells, some natural killer cells, macrophages, dendritic cells, and eosinophils impact the onset and progression of HNSCC (77). This further led us to validate the infiltration of CD4+T cells, CD8+T cells, and CD19 B cells in CBD-treated tumors compared to vehicle-treated tumors by IHC. We observed a significant increase in infiltration of CD4+T cells, CD8+T Cells, and CD19+ B cells in CBD-treated tumors as compared to control. Interestingly, we also observed that the CBD-treated tumors of HPV-positive syngeneic mice model exhibited increase in colocalization of activated form of p38 (pp38) along with CD4+T cells and CD8+T cells in the tumor microenvironment compared to vehicle-treated mice tumors by multiplex IHC analysis. Our findings are similar to a recent study defining immune triads that contain tumor-specific cytotoxic CD8+ T cells, CD4+ T cells, and antigen-presenting dendritic cells required to license tumor specific CD8+ T cells to eliminate cancer (78). This suggests that CBD may facilitate a functional immune response to tumor through multiple immune cells, facilitating coordination and activation of specific cell types. However, we would like to acknowledge that, due to limitations on the number of antibodies can be used at a time for multiplex IHC analysis, we have focused only on immune cell markers (CD4, CD8, CD45), tumor marker (pan CK), MAPK marker (pp38) and nuclei stain (DAPI). Thus, exploring the downstream targets of p38 MAPK pathway and its interaction with immune cell populations may provide additional insights about CBD mediated activation of p38 MAPK pathway and anti-tumor immune response.

This study broadens the understanding of how CBD stimulation within the tumor microenvironment modulates CD4+ T cells and CD8+ T cells by facilitating their interactions with both immune and tumor cells. In addition, a prior study by Go et al. (33), reported that CBD treatment improves the therapeutic efficacy of chemotherapeutic drugs in pre-clinical models of HPV-negative HNSCC. The effect of CBD on HNSCC has a major potential impact on public health strategies as well as therapeutic cannabinoid use and HNSCC therapy. However, in this study, we observed that CBD promotes immune infiltration and may potentially be used in combination with immunotherapy for the treatment of HNSCC. Ongoing clinical trials using a recently FDA-approved CBD (Epidiolex) (79) aim to validate CBD’s safety and efficacy in cancer therapy, however, there remains a significant gap in understanding the tumor-intrinsic effects of CBD on HNSCC, its influence on the immune microenvironment in HNSCC, and how CBD affects current HNSCC therapies through tumor microenvironment and it’s immunologic mechanisms.

In conclusion, our study suggests that CBD inhibits tumor cell proliferation in HPV-positive HNSCC by activating MAPK pathway and exhibits anti-tumor activity by modulating the CD4+T and CD8+T cells in the tumor immune microenvironment. Therefore, CBD may potentially provide a supportive role in cancer therapy through immune-mediated mechanisms. In addition, the interaction between CBD and standard care of treatments such as chemotherapy, radiotherapy, and immunotherapy require further investigation to define potential synergy and interactions. However, rigorous clinical investigation is ultimately needed to understand the efficacy and safety of CBD treatment in the context of other cancer therapies, or for therapeutic use in HPV-positive HNSCC. As such, specific cannabinoids may become an integral component of comprehensive cancer treatment regimens, offering new hope to patients with HPV-positive head and neck cancer.

## Data Availability

The raw data supporting the conclusions of this article will be made available by the authors, without undue reservation.

## Supplementary material

Supplementary Figure 1 Western blot analysis of MAPK Pathway markers post 15 minutes treatment with 10 μM of CBD in HPV-positive **(A)** UPCI: SCC154 and **(B)** UD-SCC-2 HNSCC cells.

Supplementary Figure 2 Original Western blot images of MAPK Pathway markers post 30 minutes treatment with 10 μM of CBD in HPV-positive **(A)** UPCI: SCC154 and **(B)** UD-SCC-2 HNSCC cells and 15 minutes treatment with 10 μM of CBD in HPV-positive **(C)** UPCI: SCC154 and **(D)** UD-SCC-2 HNSCC cells.

Supplementary Figure 3 CBD treatment modulates infiltration of immune cells in the Tumor Immune Micro-Environment (TIME). **(A)** Schematic representation of flow cytometric analysis of immune cell infiltration with or without treatment with CBD in immunocompetent syngeneic mouse model (wild-type) of HNSCC. The mice were injected with 1 x 10^6^ mEER cells/mice subcutaneously in the flank region, followed by I.P treatment everyday with vehicle and 10 μM of CBD from Day 6 onwards. The tumors were harvested on Day 15 and processed for flow cytometric analysis for immune cell markers. Graphical representation of cell count per mg of tumor for **(B)** dendritic cells (cDC1s), **(C)** monocytic myeloid-derived suppressor cells (M-MDSCs), **(D)** polymorphonuclear MDSCs (PMN-MDSCs), and **(E)** M2-like macrophages between the CBD-treated and vehicle-treated mice. Statistical analysis was performed by unpaired Student’s t-test [ns- non-significant].
