# Supplementary figures and images for: CBD promotes antitumor activity by modulating tumor immune microenvironment in HPV associated head and neck squamous cell carcinoma
Source: Front Immunol. 2025 May 22;16:1528520. doi: 10.3389/fimmu.2025.1528520 (PMC12137345; doi:10.3389/fimmu.2025.1528520)

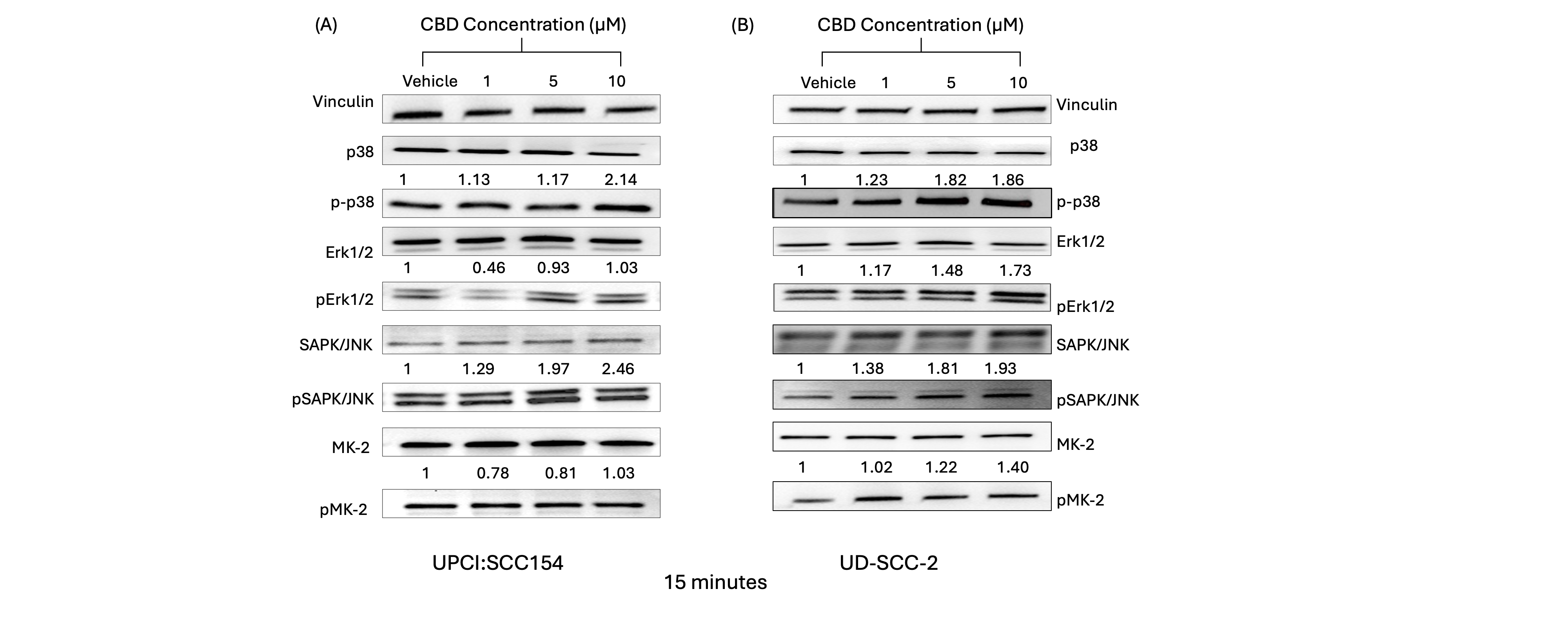

Supplement: Supplementary Figure 1 — Western blot analysis of MAPK Pathway markers post 15 minutes treatment with 10 μM of CBD in HPV-positive (A) UPCI: SCC154 and (B) UD-SCC-2 HNSCC cells. [file Image1.tiff]

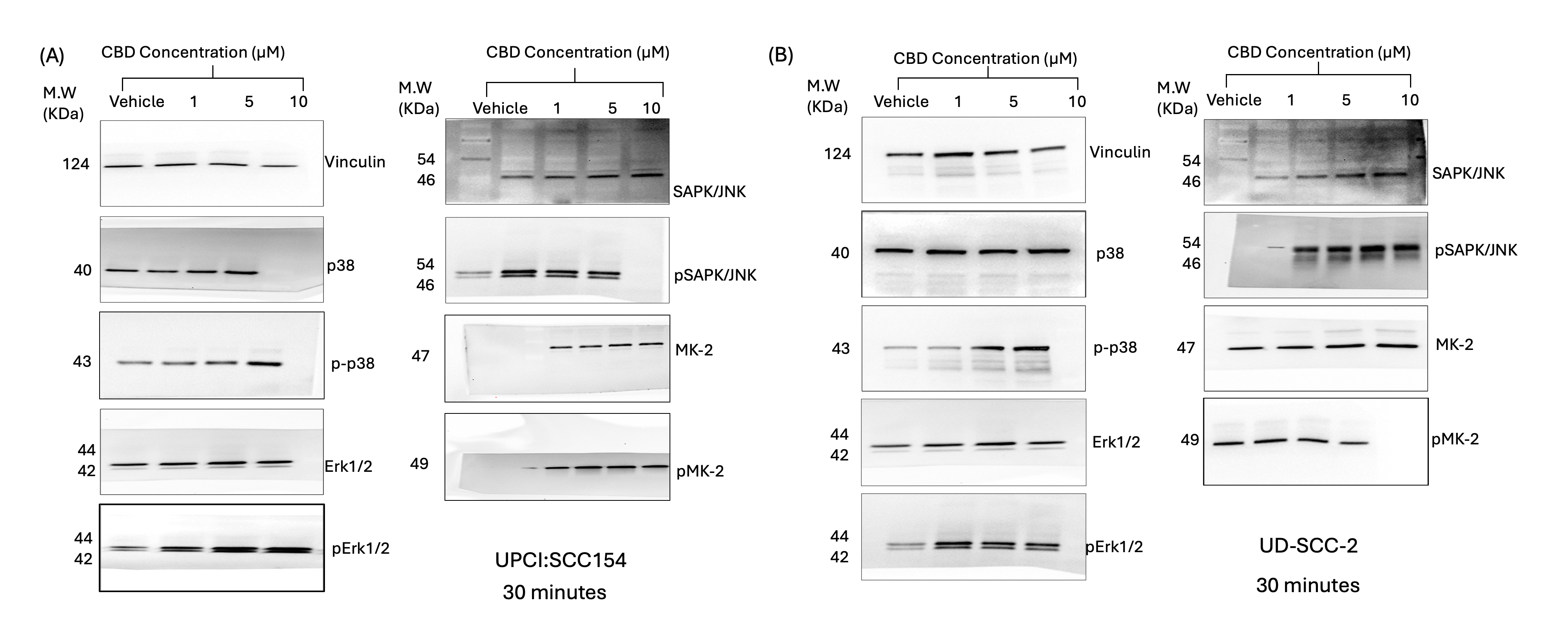

Supplement: Supplementary Figure 2 — Original Western blot images of MAPK Pathway markers post 30 minutes treatment with 10 μM of CBD in HPV-positive (A) UPCI: SCC154 and (B) UD-SCC-2 HNSCC cells and 15 minutes treatment with 10 μM of CBD in HPV-positive (C) UPCI: SCC154 and (D) UD-SCC-2 HNSCC cells. [file Image2.tiff]

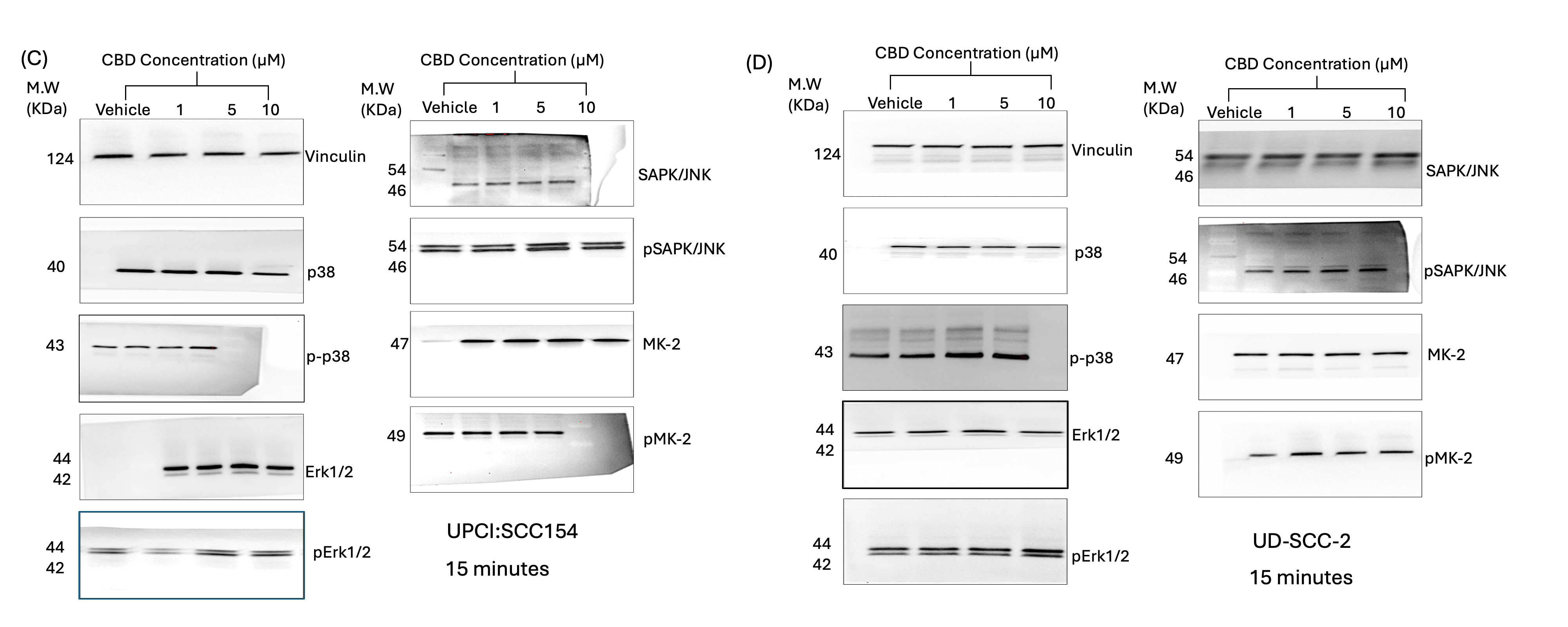

Supplement: Supplementary Figure 3 — CBD treatment modulates infiltration of immune cells in the Tumor Immune Micro-Environment (TIME). (A) Schematic representation of flow cytometric analysis of immune cell infiltration with or without treatment with CBD in immunocompetent syngeneic mouse model (wild-type) of HNSCC. The mice were injected with 1 x 10^6^ mEER cells/mice subcutaneously in the flank region, followed by I.P treatment everyday with vehicle and 10 μM of CBD from Day 6 onwards. The tumors were harvested on Day 15 and processed for flow cytometric analysis for immune cell markers. Graphical representation of cell count per mg of tumor for (B) dendritic cells (cDC1s), (C) monocytic myeloid-derived suppressor cells (M-MDSCs), (D) polymorphonuclear MDSCs (PMN-MDSCs), and (E) M2-like macrophages between the CBD-treated and vehicle-treated mice. Statistical analysis was performed by unpaired Student’s t-test [ns- non-significant]. [file Image3.tiff]

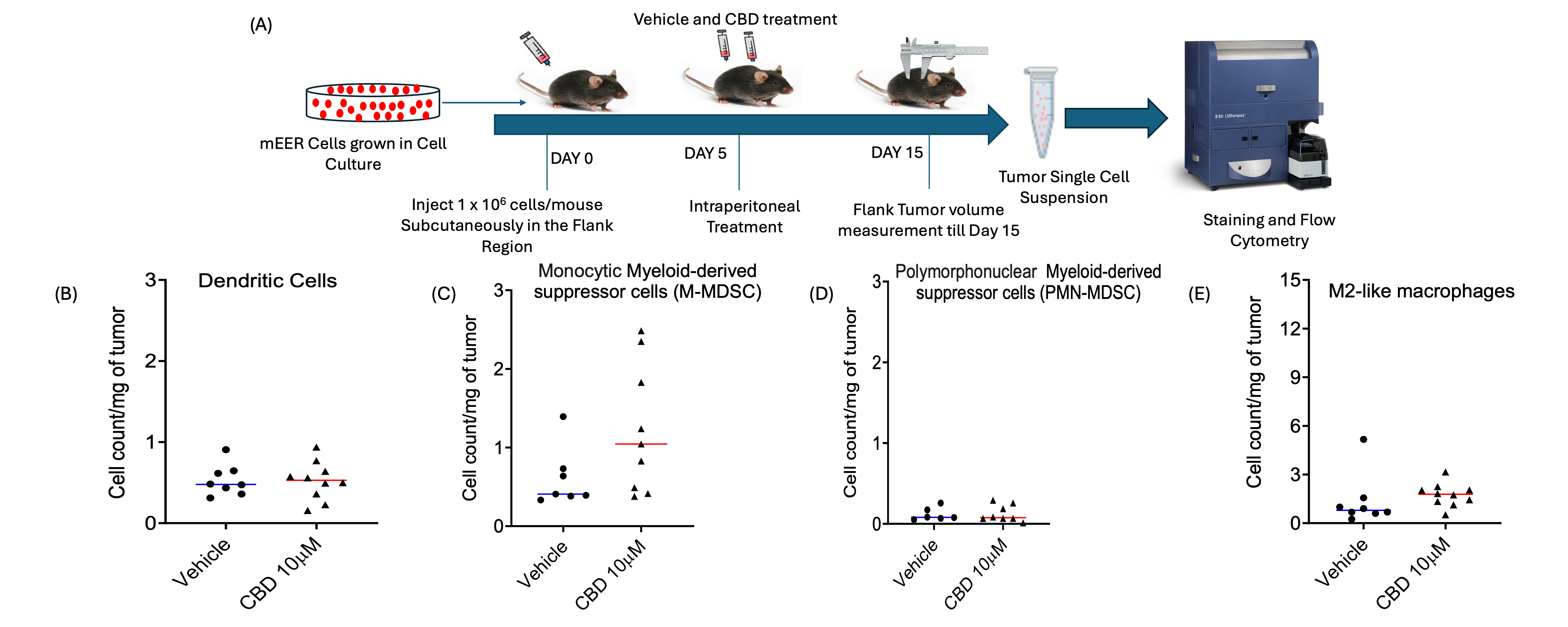

Supplement: Supplementary file 4 [file Image4.tiff]
